# Balanced motor primitive can explain generalization of motor learning effects between unimanual and bimanual movements

**DOI:** 10.1038/srep23331

**Published:** 2016-03-30

**Authors:** Ken Takiyama, Yutaka Sakai

**Affiliations:** 1 Department of Electrical and Electronic Engineering, Tokyo University of Agriculture and Technology, Tokyo, Japan; 2 Brain Science Institute, Tamagawa University, Tokyo, Japan

## Abstract

Motor learning in unimanual and bimanual planar reaching movements has been intensively investigated. Although distinct theoretical frameworks have been proposed for each of these reaching movements, the relationship between these movements remains unclear. In particular, the generalization of motor learning effects (transfer of learning effects) between unimanual and bimanual movements has yet to be successfully explained. Here, by extending a motor primitive framework, we analytically proved that the motor primitive framework can reproduce the generalization of learning effects between unimanual and bimanual movements if the mean activity of each primitive for unimanual movements is balanced to the mean for bimanual movements. In this balanced condition, the activity of each primitive is consistent with previously reported neuronal activity. The unimanual-bimanual balance leads to the testable prediction that generalization between unimanual and bimanual movements is more widespread to different reaching directions than generalization within respective movements. Furthermore, the balanced motor primitive can reproduce another previously reported phenomenon: the learning of different force fields for unimanual and bimanual movements.

In our daily life, we flexibly switch between unimanual and bimanual movements. We can adapt to novel interfaces as we unimanually manipulate a smartphone or bimanually manipulate a tablet, which are actions achieved through the motor learning of unimanual and bimanual movements. The nature of motor learning in unimanual and bimanual movements has been intensively investigated. A theoretical framework for describing motor learning is that of motor primitives^1^,^2^,^3^,^4^,^5^. In this framework, the activity of a single motor primitive *A*_*i*_ (*i* = 1, ..., *N, N* is the number of motor primitives) mimics nonlinear neural activity, and a linear combination of the nonlinear activity 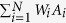 determines a motor command. Thus, each linear coefficient, *W*_*i*_ is updated to achieve desired movements. The original framework of motor primitives successfully and simultaneously reproduced nonlinear motor commands to move a nonlinear two-link arm, represented linear equations of trial-dependent changes of motor commands in unimanual movements, and demonstrated how the learning effect trained with an unimanual movement is generalized (transferred) to other unimanual movements^1^,^2^. It is also suggested that activities of motor primitives likely correspond to neural activities in motor-related brain regions^1^. The transfer of learning effects from a trained movement to other untrained movements is referred to as generalization in this manuscript. An extended framework of motor primitives has been proposed to reproduce the generalization pattern within bimanual movements^5^.

However, such distinct modeling of unimanual and bimanual movements can never explain the relationship between these two types of movements. The learning effects in bimanual reaching movements toward a fixed target or direction are partially generalized to unimanual movements^6^,^7^. That is, the learning effects trained with a bimanual movement are available not perfectly but partially in untrained unimanual movements, which is referred to as partial generalization in this manuscript. In contrast, learning effects in bimanual movements toward variable target directions sampled from eight radial directions are perfectly generalized to unimanual movements^7^. The learning effects trained with various bimanual movements are available not partially but perfectly in untrained unimanual movements, which is referred to as perfect generalization in this manuscript. The generalization pattern depends on the pattern of bimanual training; it is partial when it is trained with a single type of bimanual movement, but it is perfect when it is trained with various types of bimanual movements. The difference in generalization thus originates from the number of target directions present during the bimanual training phase, but it is not clear why the number of training target directions affects the generalization between bimanual and unimanual movements. A previous study proposed a model that consists of three different components for only unimanual, only bimanual, and both movements, respectively, and succeeded to explain the “partial” generalization on the condition of the fixed target direction^8^. However, this model cannot reproduce the perfect generalization in the condition of variable target directions. Taken together, we can conclude that a framework that explains the generalization between the two types of movements is unknown.

Here, we propose a novel model to explain the generalization of motor learning effects between unimanual and bimanual movements by incorporating the findings from neurophysiological studies into a conventional framework of motor primitives. In reaching movements, the activity of motor cortex neurons depends on the movement direction^9^, which means that neuronal activity can be considered a function of the movement direction (tuning curve). The preferred direction (PD) is defined as the movement direction in which the neuron shows its maximal activity. Some studies have observed neuronal activity in both unimanual and bimanual movements, and these studies have reported that many neurons that exhibited different tuning curve properties for unimanual and bimanual movements. For example, neural activity during unimanual and bimanual movements were modeled well by a cosine function, but the PD was modulated during unimanual and bimanual movements^10^,^11^,^12^. On the basis of these neurophysiological findings, we analytically investigated how the activity of motor primitives should be modulated between unimanual and bimanual movements to reproduce the generalization between unimanual and bimanual movements.

## Results

### General framework

**Figure 1:**
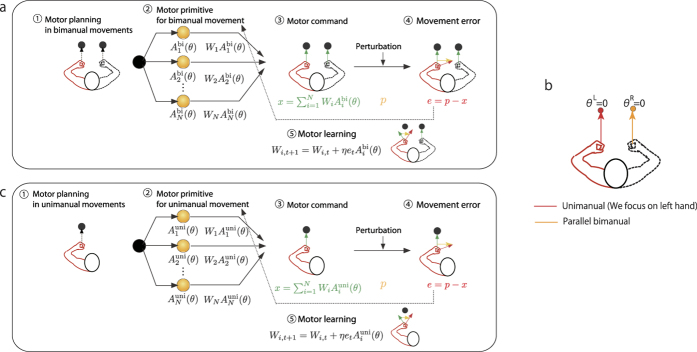
Reaching tasks and motor primitive framework. (**a**) Motor primitive framework for motor learning in bimanual movements. A given target direction (desired movement direction) *θ* determines the activity of motor primitives 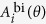 through motor planning, and the linear summation of primitive activity determines an additional motor command *x* to compensate for the movement error *e* induced by a given perturbation *p*. (**b**) In the unimanual reaching task, a target is given (red circle) for the left arm in the direction *θ*^L^ (red arrow). In the bimanual reaching task, two targets are given for the left and right arms in the directions *θ*^L^ and *θ*^R^. We focused on parallel bimanual movements in which the target directions are the same for the left and right arms (red and orange arrows, *θ*^L^ = *θ*^R^). (**c**) Motor primitive framework for motor learning in unimanual movements.

The present study focused on reaching movements towards radially distributed target directions: *θ*_1_, …, *θ*_*K*_. The target direction was randomly sampled from the *K* target directions in each trial. During each reaching movement, an unpredictable perturbation was given, such as a force field^13^ which yields a movement error *e* perpendicular to the movement direction (Fig. 1a,c). The aim of the task was to accurately reach toward a given target by generating the additional motor command *x* perpendicular to the movement direction to compensate for the movement error.

Following the original motor primitive model^1^,^2^, we assumed that the motor command *x* was a linear summation of motor primitive activities *A*_1_(*θ*), ..., *A*_*N*_(*θ*) determined by a target direction *θ* (Fig. 1a,c), i.e. 
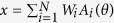
, where *W*_*i*_ determines how the *i*-th primitive contributes to the generation of a motor command. Each weight *W*_*i*_ was modified by 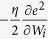 (gradient descent rule) in each trial to reduce the squared movement error *e*^2^ (see Methods), where the positive constant *η* denoted the learning rate. This framework can explain trial-dependent changes of the movement error and the generalization effects on untrained movements^1^,^2^.

The framework of motor primitives can be applied to bimanual reaching movements^3^. Throughout this study, we supposed a perturbation imposed only on the left arm (the left arm was trained, and the right arm was untrained). Therefore, the additional motor command x should be considered only for left arm movements. We considered parallel bimanual movements toward extrinsically identical directions (Fig. 1b), because previous behavioral^6^,^7^ and neurophysiological^10^,^11^ experiments focused on these types of bimanual movements. In this case, the target direction *θ* is common for two arms. The present study assumed that the activity pattern of each motor primitive for parallel bimanual movements, 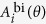, may differ from that for unimanual (left arm only) movements, 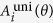. Each weight *W*_*i*_ was assumed to be common for unimanual and bimanual movements. This assumption did not mean common contributions of a weight *W*_*i*_ to unimanual and bimanual movements. The contribution of *W*_*i*_ depended on the primitive activities 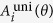 and 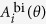. That is, each motor primitive contributed to the generation of a motor command through 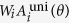 or 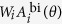.

### Generalization between unimanual and parallel bimanual movements

The learning effect of training bimanual reaching towards a fixed target direction, *K* = 1, is partially generalized to unimanual movements^6^,^7^, whereas the learning effect for *K* = 8 is perfectly generalized^7^. When the generalization is partial, the movement error increases in the trial in which bimanual movements are switched to unimanual movements. In contrast, when the generalization is perfect, movement error does not change in the trial in which bimanual movements are switched to unimanual movements. Our goal was to analytically derive the condition to reconcile the partial generalization when *K* = 1 and the perfect generalization when *K* = 8. Notably, the phrase “the perfect generalization when *K* = 8” can be indicated to be an overstatement because we are not sure whether *K* = 8 is the minimal number of targets for achieving the perfect generalization. However, a previous study^7^ reported that a perfect generalization only when *K* = 8, and there was no other evidence of the perfect generalization. We thus use this phrase in this manuscript. It was also found that the learning speed for unimanual movements was not significantly different from that for bimanual movements^14^.

Under the assumption of equivalent learning speeds between unimanual and bimanual movements, we analytically proved that the generalization is perfect if and only if 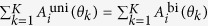 for all primitives, and it is partial otherwise (see Methods). The partial generalization for *K* = 1 was observed in the case of a certain target direction *θ*^6^,^7^, and hence, activity patterns of motor primitives for unimanual and bimanual movements toward *θ* are different. That is,


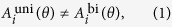


for some primitives. The perfect generalization for *K* = 8 was observed in the case of the equally distributed targets, *θ*_*k*_ = 2*πk*/8^7^. Hence, the condition for perfect generalization implied that the integrals of tuning curves might be balanced,


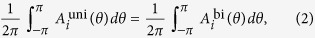


for all primitives *i* = 1, ..., *N*. This unimanual-bimanual balance indicated that, for unimanual and bimanual movements, the tuning patterns of each motor primitive might be different, but the integrals of tuning curves should be balanced. These integrals signified the mean activity of the *i*-th primitive for unimanual and parallel bimanual movements when the target direction *θ* was uniformly distributed within −*π* and *π*. Equations (1) and (2) were the only plausible candidates to reconcile the partial generalization when *K* = 1^6^,^7^, the perfect generalization when *K* = 8^7^, and the equivalent learning speeds^14^. Additionally, the weight values for unimanual and bimanual movements needed to be common


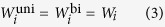


for all primitives to reconcile those phenomena.

We first derived equations (1) and (2) by assuming equation (3) (please see at *Equivalent condition to the perfect or partial generalization* section). Notably, the same equations could be achieved by assuming equation (2) and weight values that could not be common for unimanual and bimanual movements: 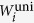 and 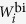. In this case, analytical calculations revealed that the conditions 
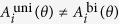
 for a certain *i* (equation (1)) and 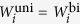 for all *i* (equation (3)) should be satisfied to reconcile the partial generalization when *K* = 1, the perfect generalization when *K* = 8, and equivalent learning speeds (detailed descriptions are provided in *Another derivation of equivalent condition to the perfect or partial generalization* in the *Methods* section). Thus, equations (1), (2), and (3) should be satisfied to reconcile the partial and perfect generalizations. Inspired mainly by equation (2), we referred to these conditions as a unimanual-bimanual balance in this manuscript.

The unimanual-bimanual balance could be simplified when the activity of each primitive was described with a common tuning curve *g*(⋅) for unimanual and bimanual movements, 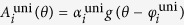, and 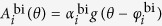, where the amplitude *α*_*i*_ and the PD *φ*_*i*_ were parameters for the *i*-th primitive, and *g*(⋅) was a periodic function, *g*(*θ*) = *g*(*θ* + 2*π*). Under the assumption of a common tuning curve, the conditions (1) and (2) are equivalent to the conditions: 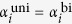 for all primitives and 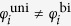 for some primitives. Namely not the amplitude but the PD was modulated by movements of the opposite arm. Notably, these analytical results were independent of the shape of the tuning curve, the distribution of PDs, and the distribution of difference in PDs between unimanual and bimanual movements.

To validate the analytical results, we numerically simulated the following three models; overlap model, amplitude modulation model, and PD modulation model.

#### Overlap model

**Figure 2:**
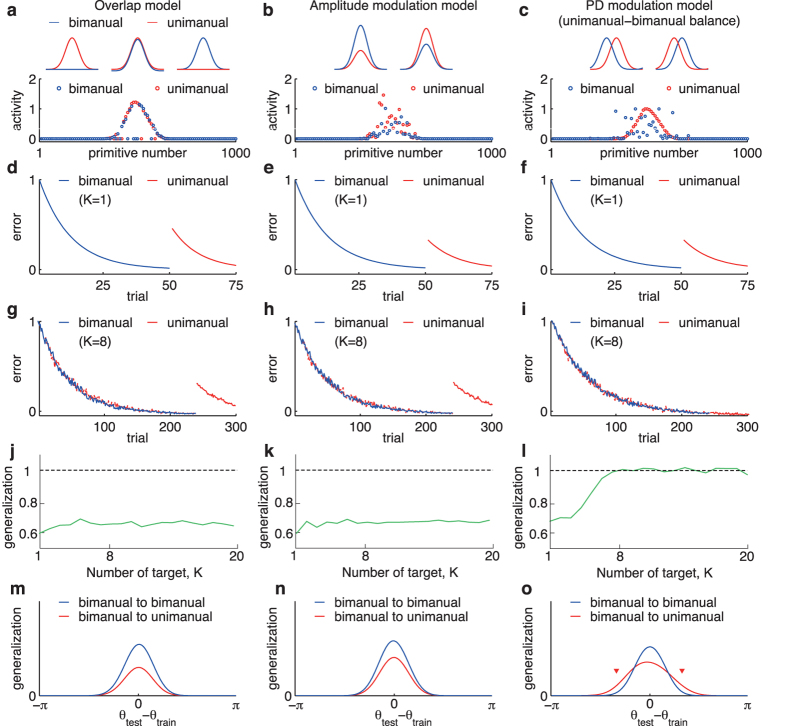
Generalization between unimanual and parallel bimanual movements in several models. (**a**–**c**) Example modulation types (upper) and an example activity pattern (lower) of primitives in the overlap model (**a**), amplitude modulation model (**b**), and PD modulation model (**c**). Red and blue lines indicate tuning curves for unimanual and parallel bimanual movements, respectively. Red and blue circles represent activities of primitives for unimanual and parallel bimanual movements when *θ* = 0. The primitives were sorted by their unimanual PDs. The circles were thinned out and shown for only 100 primitives out of 1000. (**d**–**f**) Trial-dependent changes of movement errors in the overlap (**d**), the amplitude modulation (**e**), and the PD modulation (**f**) models for a fixed target direction (*K* = 1). After 50 trials of the bimanual task (blue lines), the task switched to the unimanual task (red lines). (**g**–**i**) Trial-dependent changes of movement errors in the overlap (**g**), the amplitude modulation (**h**), and the PD modulation (**i**) models for target directions sampled from eight radial directions (*K* = 8). After 200 trials of the bimanual task (blue lines), the task switched to the unimanual task (red lines). The movement error at the first trial of the unimanual task was equivalent to that at the last trial of the bimanual task (perfect generalization) in only the PD modulation model (**i**). Red dotted lines denote learning curves with unimanual movements. (**j**–**l**) Relationships between the number of targets (*K*) and the degree of generalization at the 200th trial, which was calculated as 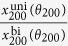. The generalization values are averaged one across 20 simulation runs. (**m**–**o**) Generalization effects after training with a parallel bimanual movement towards a target direction *θ*_train_ = 0 λ on movements towards different directions *θ*_test_ in the overlap (**m**), the amplitude modulation (**n**), and the PD modulation (**o**) models. The generalization to unimanual movements (red line) towards some target directions is larger than the generalization within bimanual movements (blue line) in the PD modulation model (hyper-generalization, indicated by red triangles in (**o**)), whereas the generalization within bimanual movements are always larger than the generalization to unimanual movements in the overlap and the amplitude modulation models (**m,n**).

A neural population related to unimanual movements overlaps with that related to bimanual movements, or some neurons are related only to unimanual or bimanual movements, whereas some are related to both^10^. Based on this neurophysiological result, a conventional computational model^8^ succeeded in reproducing a partial generalization when *K* = 1. In the present framework of motor primitives, this overlap could be modeled as parameters 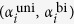, which were randomly sampled from a subset {(*α*_*i*_, 0), (*α*_*i*_, *α*_*i*_), (0, *α*_*i*_)} (three modulation types shown in the upper side of Fig. 2a). For instance, the *i*-th primitive is activated only in unimanual movements when 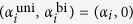. Figure 2a shows example activity pattern of primitives for a certain target direction in the overlap model.

#### Amplitude modulation model

In the overlap model, the amplitude of each primitive was modulated in an all-or-nothing manner. This constraint could be reduced as 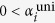, 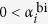, and 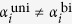. In fact, many neurons in the motor cortex exhibited tuning curves to the movement directions for both unimanual and bimanual movements in which the amplitudes were modulated^10^,^11^,^12^. We called this model the amplitude modulation model. Example modulation types and example activity patterns of primitives are presented in Fig. 2b.

#### PD modulation model

In addition to the amplitudes of tuning curves, many neurons in the motor cortex exhibited different PDs for unimanual and bimanual movements^10^,^11^,^12^. Hence, another candidate is a PD modulation model in which 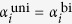 and 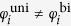, which corresponded to the unimanual-bimanual balance. Example modulation types and an activity pattern of primitives are shown in Fig. 2c.

When a constant clockwise perturbation was applied, all three models could reduce the movement errors (Fig. 2). After the task was switched from the bimanual task (blue lines) to the unimanual task (red lines), the errors that occurred during unimanual movements in all three models showed smaller values than the errors that occurred at the beginning of the bimanual task (the 1st trial of the task). Namely, the learning effects of bimanually training were transferred to untrained unimanual movements. In the condition of fixed target directions (*K* = 1), the error in the first trial of the unimanual task was larger than the error in the last trial of the bimanual task in all three models (Fig. 2d–f). This meant that the generalization effects were partial in all three models. Thus, we confirmed that all the three models reproduced the experimental results in the condition of fixed target directions (*K* = 1)^6^,^7^.

However, when the target direction in each trial was sampled from the eight radial directions (K = 8), only the PD modulation model exhibited perfect generalization (Fig. 2i), whereas the overlap and the amplitude modulation models still exhibited partial generalization (Fig. 2g,h). The red dotted lines in Fig. 2g–i denoted learning curves when trained with unimanual movements when *K* = 8. As the figures indicate, most of the curves overlapped with the curve representing trained with bimanual movements. That is, an equivalent learning speed was achieved between unimanual and bimanual movements in those models. Thus, we confirmed that only the PD modulation model could concurrently reproduce partial generalization when *K* = 1^6^,^7^, perfect generalization for *K* = 8^7^, and equivalent learning speeds between unimanual and bimanual movements^14^. Hence, these findings validated our analytical results. Figure 2j–l denote the relationship between the number of targets *K* and the degree of generalization from (trained) bimanual to (untrained) unimanual movements. As seen in the figure, a large number of targets resulted in a larger generalization only in the PD modulation model (Fig. 2l). In this case, *K* = 8 was the minimal number of targets for achieving a perfect generalization in the model. Therefore, the unimanual-bimanual balance is the key factor for explaining generalization of motor learning effects between unimanual and bimanual movements.

## Reproduction of Other Phenomena

**Figure 3:**
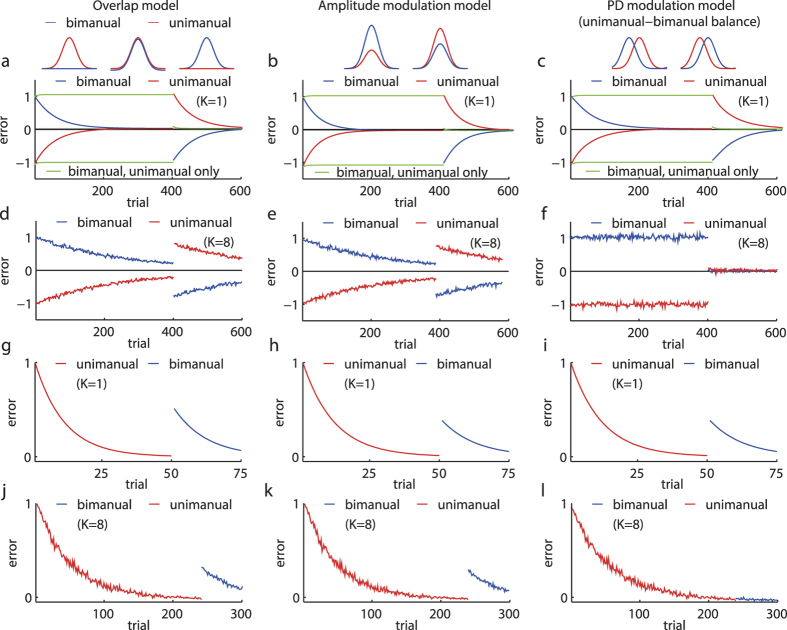
Learning of conflicting force fields and generalization from unimanual to parallel bimanual movements in several models. (**a**–**c**) Trial-dependent changes of movement errors in the overlap (**a**), the amplitude modulation (**b**), and the PD modulation (**c**) models in which conflicting force fields were learned: *p* = 1 with parallel bimanual movements (blue lines) and *p* = −1 with unimanual movements (red lines) toward a fixed target direction (*K* = 1). The two conditions were alternated in every trial. After 400 trials of the conflicting force field task, the perturbation was turned off (*p* = 0). In all models, the movement error diminished and the after effects were induced after the perturbation was turned off. Hence, all models could learn conflicting force fields. (**d**–**f**) Learning curves for conflicting force field when *K* = 8. Other settings were the same as those in (**a–c**). (**g**–**i**) Trial-dependent changes of movement errors and generalization functions in the three models in the case of the unimanual training when *K* = 1. (**j**–**l**)Trial-dependent changes of movement errors and generalization functions in the three models in the case of the unimanual training when *K* = 8.

The unimanual-bimanual balance could explain other types of phenomena. Although it is difficult to simultaneously adapt to clockwise *p* = 1 and counter-clockwise force fields *p* = −1 (conflicting force fields)^15^, the conflicting force fields can be learned with unimanual and parallel bimanual movements toward a fixed target direction (*K* = 1)^6^. For example, a clockwise force field was learned with unimanual movements and a counter-clockwise force field was learned with bimanual movements, and vice versa. The PD modulation model could learn the conflicting force fields during unimanual and parallel bimanual movements toward a fixed target direction (*K* = 1) (Fig. 3c), which was also true for the overlap and the amplitude modulation models (Fig. 3a,b). These models could also reproduce the difficulty of learning a conflicting force field with only unimanual or bimanual movements (green lines in Fig. 3a–c). Because the motor primitives exhibited different recruitment patterns for unimanual and bimanual movements when *K* = 1 (Fig. 2c), a certain population could learn to increase the strength of its contribution to a clockwise (counter-clockwise) force field to exceed the contribution of other populations, and a different population could learn to strengthen its contributions to a counter-clockwise (clockwise) force field. In contrast, the unimanual-bimanual balance predicted that subjects could not learn the conflicting force field when *K* = 8 (Fig. 3f). In this case, the generalization from bimanual to unimanual movements was perfect. Each motor primitive for unimanual movements needed to learn both the clockwise and counter-clockwise force field simultaneously, which caused an averaged movement error of 0 and no motor learning. In other words, the conflicting force field could be learned when the generalization was partial, but it could not be learned when the generalization was perfect. In addition, the PD modulation model could reproduce a partial generalization from unimanual training to bimanual movements when *K* = 1 (Fig. 3f)^6^, as well as the overlap and the amplitude modulation models (Fig. 3d,e). Furthermore, the perfect generalization could be observed from (trained) unimanual to (untrained) bimanual movements if the unimanual-bimanual balance was satisfied (Fig. 3l). Because learning in the presence of a conflicting force field when *K* = 8 and the perfect generalization from unimanual to bimanual movements when *K* = 8 have not been reported in previous studies, these were our predictions based on the unimanual-bimanual balance.

## Discussion

We proposed the unimanual-bimanual balance based on a motor primitive framework to explain the generalization of motor learning effects between unimanual and bimanual movements. The unimanual-bimanual balance (equations (1), (2), and (3), or PD modulation) successfully reproduced generalization patterns between unimanual and bimanual movements. The partial generalization on the condition that the training target direction is fixed^6^, and the perfect generalization on the condition that the training target is sampled from the eight radial directions in parallel bimanual movements^7^ (Fig. 2f,i). The unimanual-bimanual keeps the ability to explain other phenomena of motor learning in unimanual and bimanual movements: learning under a conflicting force field in unimanual and bimanual movements (Fig. 3c), the partial generalization from unimanual training to bimanual movements toward a fixed target direction^6^ (Fig. 3i). The present framework of unimanual and bimanual primitives is symmetric for unimanual and bimanual movements. Therefore, the same conclusion regarding the generalization from bimanual to unimanual movements can be applied for the generalization from unimanual training to bimanual movements (Fig. 3i,l).

### Comparison with conventional models

Conventional frameworks of motor primitives successfully reproduced generalization patterns when the kinematics of reaching movements (e.g., target direction) changed in unimanual^1^,^2^,^4^ or bimanual movements^5^. However, those models could explain the generalization pattern *only* in unimanual movements or those *only* in bimanual movements. In the model for unimanual movements, some motor primitives were activated when the desired movement direction was 0°, but it was unclear how these primitives were activated in bimanual movements. In the model of bimanual movements, some motor primitives were activated when the desired movement direction for the left and right arms were 0°. However, it remains unknown how these primitives are activated in unimanual movements. One potential explanation is an overlap. A partial generalization between unimanual and bimanual movements could be reproduced by an overlap of a neural population for unimanual movements and that for bimanual movements^8^. In that model, the learning effects were embedded into both non-overlapped and overlapped neural populations. Learning effects in the overlapped neural population were shared between unimanual and bimanual movements, resulting in partial generalization between those movements. Nevertheless, based on the overlap model, the generalization was always partial independent of the number of targets, which contradicted to the results of behavioral experiments^7^ (Fig. 2g). Taken together, the unimanual-bimanual balance was the first framework that could explain generalization of learning effects between unimanual and bimanual movements.

### Relationships between unimanual and bimanual movements

The unimanual-bimanual balance might suggest a novel relationship between unimanual and bimanual movements regarding the specialization and generalization of learning. In Fig. 2o, we note the hyper-generalization as a testable prediction of the unimanual-bimanual balance. The generalization pattern within bimanual movements was higher but narrower than that from bimanual to unimanual movements, which meant that the generalization after training with bimanual movements was restricted only to the bimanual movements with kinematics that were similar to trained movements. In other words, specialization was prioritized over generalization. In contrast, the generalization pattern from bimanual to unimanual movements was lower but wider than that within bimanual movements, leading to a partial generalization to the same target direction but widespread generalization to different target directions. Generalization was prioritized over specialization. The unimanual-bimanual balance supported the compatibility of specialization and generalization in a single primitive framework.

### Relationship with neural activity

When *A*_*i*_(*θ*) was modeled as *α*_*i*_*g*(*θ* − *φ*_*i*_), the balanced motor primitive was equivalent to the PD modulation, 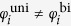. Although neural implementation of the PD modulation or unimanual-bimanual balance remains unclear, a candidate of its implementation was sparse corpus callosum connectivity between neurons in right and left motor cortices^16^, i.e., a small portion of neurons were connected through the corpus callosum. Not sparse but all-to-all callosal connections do not cause PD modulation^16^ or they cause only a PD shift^17^, 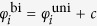 for all *i*, where *c* is a common constant for all *i*, and the shift does not seem biologically plausible. Further, an anatomical study reported that the callosal connections between the motor cortices are sparse^18^. Thus, sparse callosal connections can be a candidate to implement PD modulation, or a unimanual-bimanual balance.

The PD modulation seemed to be contradictory to a previous neurophysiological result: both the PD and amplitude were modulated between unimanual and bimanual movements^11^. However, previous studies focused on parallel and opposite movements (target directions for left, *θ*^L^, and right arm, *θ*^R^, are related to each other as |*θ*^L^ − *θ*^R^| = 180°), which is in contrast to the current study where we focused only on parallel bimanual movements inspired by previous behavioral experiments^6^,^7^. These differences could have caused the discrepancy. Further, the PD modulation is just a simple solution of the unimanual-bimanual balance with assuming a common tuning curve. As long as it is balanced, the amplitude modulation is also allowed if the shape of the tuning curve is also modulated. Hence, the unimanual-bimanual balance can be consistent with the neurophysiological result.

Several studies have previously discussed a relationship in the neural activities between unimanual and bimanual reaching movements by decomposing those movements into two parts: motor planning and execution^11^,^16^. The motor planning phase is defined as the phase in which a target position or a desired movement direction is determined before the onset of a reaching movement. In the framework of motor primitive, the recruitment pattern of primitives is determined in motor planning. Motor execution is defined as the phase around the onset of the movements. In motor primitive framework, motor commands *x* are determined during motor execution. Although the difference in neural activity between unimanual and bimanual movements is mainly caused by the corpus callosum, it remains unclear whether the corpus callosum affects neural activity only in motor planning, only in motor execution, or in both motor planning and execution. Some computational studies suggested that neural activity was significantly modulated mainly in motor planning^11^,^16^. Our results also support that the corpus callosum significantly affects neural activity mainly in motor planning. The unimanual-bimanual balance determines the recruitment pattern of motor primitives in motor planning, which suggests that the difference in neural activity between unimanual and bimanual movements can be observed mainly in motor planning.

### Limitations and future work

Although the present study analytically revealed a relationship between unimanual and bimanual movements by focusing on parallel bimanual movements, the balanced motor primitive needs to be extended to include motor learning in various types of bimanual movements. The perfect generalization from bimanual to unimanual movements when *K* = 8 could be observed in the case of not only parallel bimanual movements but also symmetrical bimanual movements (the sign of target angle for left arm was opposite to that for right arm, e.g., *θ* = 30° for left arm and *θ* = −30° for right arm)^7^. This result could lead to the unimanual-bimanual balance (equations (1) and (2)) both for parallel and symmetrical bimanual movements. A previous study extensively investigated generalization within various types of bimanual movements^5^. Another previous study reported that learning speed is not significantly different between unimanual movements and bimanual movements when the target directions were random and independent for the left and right arms^14^. The partial transfer when *K* = 1 was likely to be specialized for bimanual movements because it could not be observed with simultaneous movements of unimanual or ankle movements^6^. An extended version of the balanced motor primitive framework needs to be proposed to simultaneously reproduce these results. Further, we proposed that motor primitives whose activities were determined on the basis of a prospective (predicted) movement error could reproduce several motor-learning-related phenomena within a unified manner^3^. How to reconcile the unimanual-bimanual balance and the prospective error model is another future work.

## Methods

### Learning rule and generalization function

The movement error feedback *e* perpendicular to the movement direction was supposed to be proportional to the difference between the given perturbation *p* (*p* was set to 1 or −1 in this study) and the generated motor command *x, e* ∝ *p* − *x*. The motor command *x* to be generated for a target direction *θ* was described as 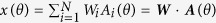, where the vectors represented ***W*** = (*W*_1_, ..., *W*_*N*_) and ***A***(*θ*) = (*A*_1_(*θ*), ..., *A*_*N*_(*θ*)) respectively, and the dot · denoted the inner product of vectors. The gradient of the square error *e*^2^ for a weight *W*_*i*_ was obtained as 
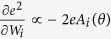
. Thus the gradient learning rule in trial *t* was obtained as ***W***_*t*+1_ = ***W***_*t*_ + *ηe**A***(*θ*), where error feedback *e* and target direction *θ* were given in trial *t*. A positive constant *η* represented the learning rate. This learning rule enabled us to predict how learning effects at the *t*-th trial with *θ* were available at the (*t* + 1)-th trial with *θ*′, where *θ*′ (that might be different from *θ*) was a target direction presented in the trial. Multiplying ***A***(*θ*′) by the learning rule, we obtained *x*_*t*+1_(*θ*′) = *x*_*t*_(*θ*′) + *ηe*_*t*_***A***(*θ*′) ⋅ ***A***(*θ*), where *x*_*t*_(*θ*′) was the motor command to be generated for a target *θ*′. Because the motor command *x*_*t*_(*θ*′) was unobservable, it indicated a predicted value. When ***A***(*θ*) ⋅ ***A***(*θ*′) = 0, *x*_*t*+1_(*θ*′) = *x*_*t*_(*θ*′), indicating that the learning effects at the *t*-th trial with *θ* were not available at the next trial *θ*′. In contrast, when ***A***(*θ*) ⋅ ***A***(*θ*′) = ***A***(*θ*) ⋅ ***A***(*θ*), *x*_*t*+1_(*θ*′) − *x*_*t*_(*θ*′) = *x*_*t*+1_(*θ*) − *x*_*t*_(*θ*), indicating that the learning effects at the *t*-th trial with *θ* were perfectly available at the next trial with *θ*′. We referred to this perfect availability of the learning effects as perfect generalization. The case when *θ*′ = *θ* was a self-evident case of perfect generalization. Further, when ***A***(*θ*) ⋅ ***A***(*θ*′) < ***A***(*θ*) ⋅ ***A***(*θ*), *x*_*t*+1_(*θ*′) − *x*_*t*_(*θ*′) < *x*_*t*+1_(*θ*) − *x*_*t*_(*θ*), indicating that learning effects at the *t*-th trial with *θ* were partially available at the next trial with *θ*′. We referred to this partial availability of learning effects as partial generalization. The generalization of learning was thus determined by the factor ***A***(*θ*′) ⋅ ***A***(*θ*). The factor for the same direction ***A***(*θ*) ⋅ ***A***(*θ*) = |***A***(*θ*)|^2^ determines the learning speed. This could be applied for generalization between unimanual and parallel bimanual movements. The generalization from unimanual training to parallel bimanual movements was determined by the factor ***A***^bi^(*θ*′) ⋅ ***A***^uni^(*θ*), and by the factor ***A***^uni^(*θ*′) ⋅ ***A***^bi^(*θ*), and vice versa. For example, when ***A***^uni^(*θ*) ⋅ ***A***^bi^(*θ*) < ***A***^bi^(*θ*) ⋅ ***A***^bi^(*θ*), 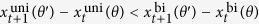, indicating that the partial generalization from bimanual to unimanual movements. When ***A***^uni^(*θ*) ⋅ ***A***^bi^(*θ*) = ***A***^bi^(*θ*) ⋅ ***A***^bi^(*θ*), 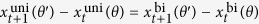, indicating that the perfect generalization from bimanual to unimanual movements.

### Equivalent condition to the perfect or partial generalization

The generalization of the learning effects in each trial for bimanual movements toward multiple training directions was determined by the average over all training target directions, 
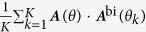
. The learning effect and the generalization effect were experimentally evaluated as the average over the test target directions which were common to the training directions^7^. Therefore, the average effects of bimanual learning and generalization to unimanual movements were determined by 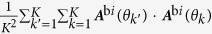 and 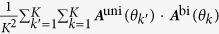 respectively. Comparison between these two values determined whether the generalization from bimanual to unimanual movements was perfect or partial, i.e., when 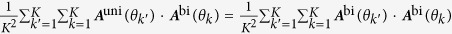, 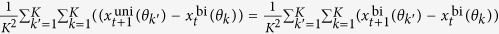, indicating a perfect generalization from bimanual to unimanual movements.

Under the assumption of equivalent learning speeds, 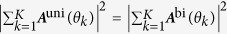, we obtained


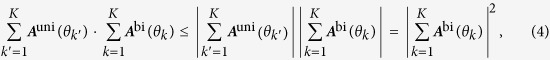


using the Cauchy-Schwartz inequality. The equality condition of the Cauchy-Schwartz inequality was 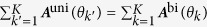. Thus, under the assumption of equivalent learning speeds, the perfect generalization occurred if and only if


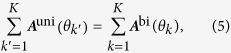


and otherwise, the partial generalization occurred, i.e., partial generalization when *K* = 1 occurs if and only if ***A***^uni^ ≠ ***A***^bi^.

### Another derivation of equivalent condition to the perfect or partial generalization

To validate our conclusion, we derived the condition to reconcile a partial and perfect generalization from another perspective. In the above derivations, we first assumed that weight values ***W*** were common for unimanual and bimanual movements. In this section, we assumed a normalization of each motor primitive activity, 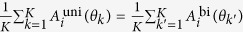, and weight values that could be different between unimanual and bimanual movements, 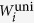 and 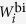. In this case, we needed to define a parameter *β* that determined how learning effects are generalized between unimanual and bimanual movements. Generalizations within bimanual and from bimanual to unimanual movements were determined by 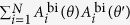 and 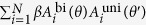, respectively.

Perfect generalization could be reproduced if and only if 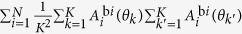 = 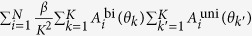. On the basis of the assumption 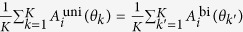, the condition to reproduce perfect generalization could be written as


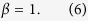


In this condition, learning rules for 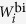 and 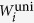 with bimanual training could be written as 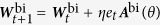 and 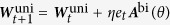, respectively. Similarly, learning rules with unimanual training could be written as 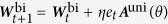 and 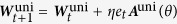. With the same initial values 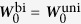, these learning rules could lead ***W***^bi^ = ***W***^uni^.

Partial generalization when *K* = 1 could be reproduced when


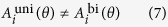


for a certain *i*.

### Prediction of hyper-generalization

The partial generalization for *K* = 1 and the perfect generalization for *K* = 8 led to the prediction of the hyper-generalization:


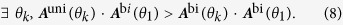


This was because the summation of the left hand, 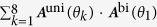, equaled the summation of the right hand, 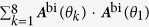, because the condition of the perfect generalization (5) for *K* = 8, though the first term ***A***^uni^(*θ*_1_) ⋅ ***A***^bi^(*θ*_1_) < ***A***^bi^(*θ*_1_) ⋅ ***A***^bi^(*θ*_1_) since the partial generalization for *K* = 1.

This prediction meant that the generalization between the different types of movements (from bimanual to unimanual) was larger than the generalization within the same type of movements (from bimanual to bimanual) for a certain test target direction *θ*_*k*_.

### Procedure to demonstrate behavioral task

The perturbation imposed on the left arm was set to be constant (assumed as clockwise perturbation) (Fig. 2d–i). In the simulations for *K* = 1 (Fig. 2d–f), the fixed target direction was *θ*_1_ = 0. After 50 trials of the bimanually reaching task, the task was changed to the unimanual task. The perturbation was still consistent in the unimanual task. In the simulations for *K* = 8 (Fig. 2g–i), a target direction was sampled independently from *θ*_*k*_ = *πk*/4 − *π* (*k* = 1, ..., 8) in each trial. After 200 trials of the bimanual reaching task (parallel or symmetric), the task switched to the unimanual task. The perturbation was still consistent in the unimanual task. In Fig. 3g–l, the same procedures were used with unimanual movements followed by bimanual movements.

In Fig. 3d–f, learning of a conflicting force field was simulated by assuming unimanual movements with *p* = −1 and bimanual movements with *p* = 1 alternately.

### Motor primitives for Fig. 2

The number of motor primitives *N* was set to 1000. We used a truncated Gaussian tuning curve^1^,^2^: 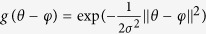, where 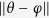 was a periodic function 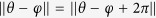 such that ||*θ* − *φ*|| = *θ* − *φ* for −*π* ≤ *θ* − *φ* < *π*. The PD *φ* was sampled from a uniform distribution in the range [−*π, π*]. The tuning width *σ* was set to *π*/10. The learning rate was set as *η* = 1/*N* = 0.001. In the simulation of the overlap model, the amplitude pair 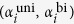 of each primitive was randomly sampled from {(*α*, 0),(0, *α*),(*α, α*)} with the even probability 1/3. The constant amplitude was set as 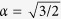. In the simulation of the amplitude modulation model, the amplitudes 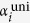 and 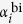 were independently sampled from a gamma distribution of the mean 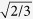 and variance 1/3. In the simulation of the PD modulation model, the amplitudes were set as 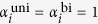. The preferred direction (PD) pair 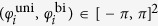 of each primitive was sampled from the probability distribution 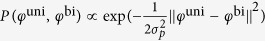, where *σ*_*p*_ = 1.5*σ*. These parameters were set such that the expected learning speeds *η*|**A**(*θ*)|^2^ for the three models are equal. In the all simulations, the initial values were set as *W*_*i*_ = 0 for all *i*.

## Additional Information

**How to cite this article**: Takiyama, K. and Sakai, Y. Balanced motor primitive can explain generalization of motor learning effects between unimanual and bimanual movements. *Sci. Rep.* **6**, 23331; doi: 10.1038/srep23331 (2016).
